# Homogeneous Aptasensor with Electrochemical and Electrochemiluminescence Dual Detection Channels Enabled by Nanochannel-Based Probe Enrichment and DNase I Cleavage for Tumor Biomarker Detection

**DOI:** 10.3390/molecules30030746

**Published:** 2025-02-06

**Authors:** Jiong Gao, Shiyue Zhang, Fengna Xi

**Affiliations:** 1 Shanxi Bethune Hospital, Shanxi Academy of Medical Sciences, Third Hospital of Shanxi Medical University, Tongji Shanxi Hospital, Taiyuan 030032, China; gaojiong@sxbqe.com.cn; 2 School of Chemistry and Chemical Engineering, Zhejiang Sci-Tech University, Hangzhou 310018, China; 202120104186@mails.zstu.edu.cn

**Keywords:** homogeneous aptasensor, electrochemical, electrochemiluminescence, dual detection channels, DNase I cleavage

## Abstract

Homogeneous aptasensors that eliminate the need for probe labeling or immobilization hold significant potential for the rapid detection of tumor biomarkers. Herein, a homogeneous aptasensor with electrochemical (EC) and electrochemiluminescence (ECL) dual detection channels was developed by integrating nanochannel-based probe enrichment and DNase I cleavage for selective detection of the tumor biomarker, carbohydrate antigen 125 (CA125). A two-dimensional (2D) composite probe was prepared by assembling the CA125-specific aptamer and the cationic probe tris(2,2′-bipyridyl)Ru(II) (Ru(bpy)_3_^2+^), which exhibited both EC and ECL properties, onto graphene oxide (GO) nanosheets (Ru(bpy)_3_^2+^/Apt@GO). A vertically ordered mesoporous silica film (VMSF) with ultrasmall, uniform, and vertically aligned nanochannel arrays was rapidly grown on the inexpensive and disposable indium tin oxide (ITO) electrode, forming the detection interface. Due to the size exclusion effect of the ultrasmall nanochannels in VMSF, the Ru(bpy)_3_^2+^/Apt@GO probe was unable to penetrate the nanochannels, resulting in no detectable Ru(bpy)_3_^2+^ signal on the electrode. Upon specific recognition of CA125 by the aptamer, an aptamer-CA125 complex was formed and subsequently detached from GO. DNase I then cleaved the aptamer-CA125 complex, releasing CA125 and allowing Ru(bpy)_3_^2+^ to dissociate into the solution. This enzymatic cleavage enabled CA125 to re-enter the binding cycle, amplifying the release of Ru(bpy)_3_^2+^ into the solution. The electrostatic adsorption of the cationic Ru(bpy)_3_^2+^ by VMSF significantly enhanced both the EC and ECL signals. The constructed aptasensor exhibited a linear EC detection range for CA125 from 0.1 U/mL to 100 ng/mL, with a limit of detection (LOD) of 91 mU/mL. For ECL detection, CA125 was detected over a range from 0.001 to 100 U/mL, with a LOD as low as 0.4 mU/mL. The developed aptasensor demonstrated excellent selectivity and was successfully applied to the dual-mode EC/ECL detection of CA125 in fetal bovine serum samples.

## 1. Introduction

Cancer poses a serious threat to human health. Tumor marker detection plays an important role in assisting early cancer screening and evaluating treatment effectiveness [1]. As the most frequently used tumor biomarker, carbohydrate antigen 125 (CA125) is related to lung cancer, gastrointestinal cancer, and ovarian cancer [2]. Developing a sensitive, accurate, and easy-to-operate method for CA125 detection is of great significance. Currently, the commonly used methods for CA125 detection include enzyme-linked immunosorbent assay (ELISA), surface plasmon resonance (SPR), fluorescence, electrochemical methods, and electrochemiluminescence (ECL) techniques [3,4,5,6]. Electrochemical methods demonstrate great potential due to their high sensitivity, excellent selectivity, simple instrumentation, rapid response time, and easy miniaturization [7,8,9,10]. Meanwhile, the ECL method offers advantages such as low background noise and a wide dynamic range [11,12,13,14,15]. The EC/ECL dual-mode detection, which provides electrochemical and ECL dual detection channels, integrates the strengths of both techniques [16,17,18]. Constructing an EC/ECL dual-mode detection platform using probes with both electrochemical and ECL properties is highly desirable.

Aptasensors, based on the specific recognition of target molecules by aptamers, show considerable potential in tumor biomarker detection [19,20,21]. Aptamers, which are single-stranded RNA or DNA molecules, could bind to target molecules (such as proteins or small molecules) to form aptamer-target complexes, exhibiting high selectivity and affinity [22,23]. Compared to antibodies, aptamers offer advantages such as high stability, easy synthesis, and low cost, making them ideal recognition elements for biosensors [24]. However, traditional aptasensors often employ heterogeneous sensing modes, requiring immobilization of recognition aptamers on the electrode surface, which is typically time-consuming [25,26,27]. Some sandwich-type aptasensors also require aptamer labeling. In contrast, the homogeneous aptasensor simplifies sensing by eliminating the need for aptamer immobilization or labeling [28]. This approach also has advantages, including reduced cost, simplified experimental procedure, and saved time. In addition, the binding efficiency between the target and aptamer probes is relatively high since aptamer-target recognition occurs in solution. Thus, developing convenient, easy-to-operate, low-cost, and highly sensitive homogeneous aptasensors has great promise for rapid detection of CA125.

Integrating aptamers with nanomaterials to prepare nanorecognition probes is an effective strategy for constructing high-performance biosensing systems [29,30]. Among these materials, graphene oxide (GO) has attracted significant attention [31,32,33]. As a two-dimensional (2D) material with sp^2^-hybridized carbon atoms in a single atomic layer, GO exhibits high surface area and good biocompatibility [34]. Its large surface area provides numerous sites for aptamer immobilization, which effectively enhances the sensor’s sensitivity. Additionally, the growth of vertically ordered mesoporous silica nanofilms (VMSF) on electrode surfaces has emerged as an important method for fabricating high-performance modified electrodes [35,36,37,38]. The ultrasmall nanochannel array endows VMSF-modified electrodes with unique properties. On the one hand, the nanochannel size of 2–3 nm in VMSF allows small electrochemical probes to access the electrode surface while excluding large molecules due to size effects [39,40,41,42]. On the other hand, after the dissociation of silanol groups (Si-OH, p*K*_a_~2) on the VMSF surface, the surface acquires a negative charge, significantly enhancing the electrostatic enrichment of cationic electrochemical probes and greatly amplifying the signal [43,44,45,46]. Therefore, combining 2D probes with VMSF-modified electrodes demonstrates great potential for constructing homogeneous aptasensors for bioanalysis.

In this work, a homogeneous aptasensor based on VMSF-modified electrodes was developed for CA125 detection using EC/ECL dual channels. Low-cost, disposable indium tin oxide (ITO) electrodes were used, with a nanochannel array film of VMSF grown on the surface. By π-π interaction and electrostatic forces, CA125 aptamers were easily complexed with electrochemical/electrochemiluminescence probe Ru(bpy)_3_^2+^ and immobilized on graphene oxide (GO), creating a two-dimensional (2D) nanocomposite probe (Ru(bpy)_3_^2+^/CA125 Apt@GO). The size exclusion effect of the ultrasmall nanochannels in the VMSF blocked Ru(bpy)_3_^2+^/Apt@GO from entering the channels, preventing the detection of the Ru(bpy)_3_^2+^ signal on the electrode. When the aptamer recognized the target CA125 and formed the aptamer-CA125 complex, the complex detached and was cleaved by DNase I, releasing CA125 and causing Ru(bpy)_3_^2+^ to fall into the solution. Through cyclical cleavage, CA125 continuously entered the binding process, further increasing the release of Ru(bpy)_3_^2+^ into the solution. The electrostatic adsorption of Ru(bpy)_3_^2+^ on VMSF significantly enhanced both EC and ECL signals, enabling sensitive detection of CA125. This sensor exhibited high sensitivity and was suitable for rapid detection of CA125.

## 2. Results and Discussion

### 2.1. Strategies for Construction of Homogeneous Aptasensor and EC/ECL Dual-Mode Detection

**Figure 1:**
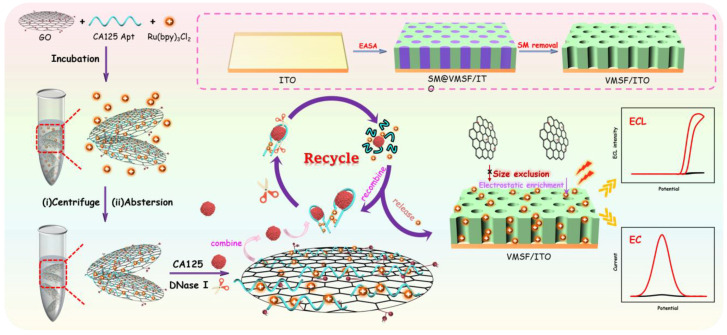
Schematic illustration for the fabrication of the homogeneous aptasensor by integrating the exclusion properties of SNF toward 2D materials, the enrichment toward positively charged probes, and the cyclic cleavage activity of nucleases for dual-mode EC and ECL detection of CA125.

In this study, the exclusion properties of SNF toward two-dimensional (2D) materials, the enrichment of positively charged probes, and the cyclic cleavage activity of nucleases were synergistically integrated to develop a homogeneous aptasensor for dual-mode electrochemical (EC) and electrochemiluminescence (ECL) detection of CA125. As shown in Figure 1, the nanochannel array film (SNF/ITO) was rapidly integrated on the surface of the ITO electrode using the electro-assisted self-assembly (EASA) method. Graphene oxide (GO) was employed as a 2D nanocarrier to simultaneously immobilize Ru(bpy)_3_^2+^ and the recognitive aptamer (Apt), forming a 2D composite probe (Ru(bpy)_3_^2+^/Apt@GO) that combines both signal molecules and recognition elements. In solution, the GO and CA125 aptamer (Apt) stack via π–π interactions. Due to its strong negative charges, the positively charged probe Ru(bpy)_3_^2+^, which possessed both EC and ECL signal properties, was tightly adsorbed onto the GO sheets or within the grooves of the CA125 aptamer via electrostatic interactions, resulting in the formation of the 2D nanocomposite probe.

In the presence of the target analyte CA125 and DNase I in the detection solution, the aptamer bound to CA125 to form an aptamer-target complex, which detached from the GO sheets and was exposed to the bulk solution, no longer protected by GO. Subsequently, DNase I cyclically cleaved the aptamer, releasing the target molecule CA125 into the next recognition process. Simultaneously, the enzymatic hydrolysis of the aptamer complex by DNase I allowed CA125 to interact further with unbound aptamers, thereby achieving additional release of Ru(bpy)_3_^2+^. Consequently, the enzymatic cleavage facilitated the cyclic recognition of CA125 by the aptamer and the repetitive release of Ru(bpy)_3_^2+^. At the same time, the nanochannels of the SNF significantly enriched the positively charged probe Ru(bpy)_3_^2+^, which possessed both EC and ECL signals, enabling dual-mode detection of CA125 with enhanced sensitivity.

### 2.2. Characterization of VMSF and VMSF-Modified ITO Electrode

**Figure 2:**
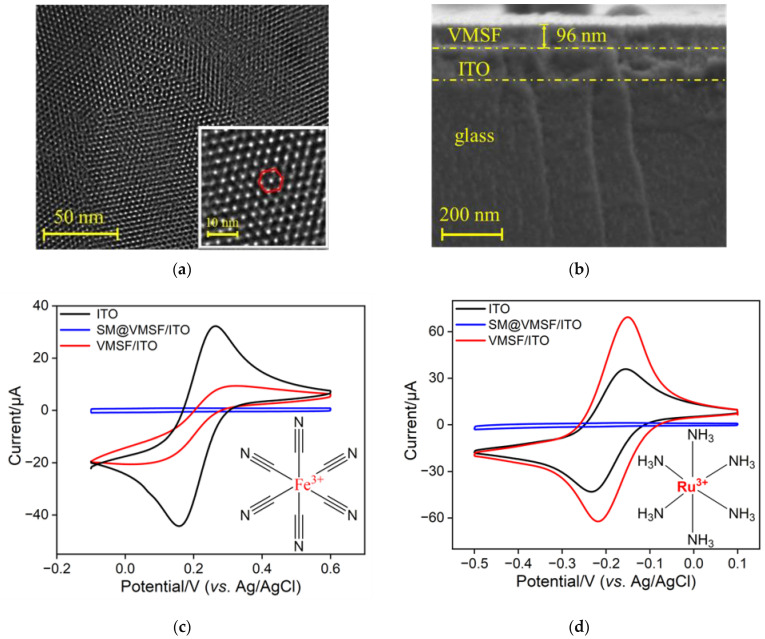
(**a**) Top-view TEM image of VMSF. Inset is the corresponding high-resolution TEM image. The red markings in the inset represented the hexagonal structure composed of nanochannels. (**b**) SEM image of the cross-sectional of VNSF/ITO electrode. (**c**,**d**) CV curves recorded on bare ITO (black line), SM@VMSF/ITO containing micelles (blue line), and VMSF/ITO with open nanochannel (red line) electrodes in solutions containing different probes. The electrolyte solution was 0.05 M KHP (pH 7.4) containing 0.5 mM of Fe(CN)_6_^3−^ (**c**), or Ru(NH_3_)_6_^3+^.

The morphology of VMSF and VMSF-modified electrodes was characterized using TEM and SEM. Figure 2a shows the TEM image of the VMSF surface, revealing an ordered nanochannel structure. Analysis using Image J software (V. 1.8.0) determined the nanochannel diameter of VMSF to be approximately 2.3 nm. The inset high-resolution TEM image (inset in Figure 2a) further illustrates a uniformly distributed hexagonal arrangement. Figure 2b displays the cross-sectional view of the VMSF/ITO electrode, clearly showing distinct interfacial layers. From top to bottom, the layers include the VMSF layer, the indium tin oxide (ITO) and the glass substrate of ITO conductive glass. The thickness of the VMSF layer was measured to be 96 nm.

The integrity and permeability of the VMSF films were investigated using CV with two standard redox probes. In the experiments, Fe(CN)_6_^3−^ and Ru(NH_3_)_6_^3+^, which carried negative and positive charges, were used as standard probes. The ITO electrode, SM@VMSF/ITO electrode containing micelles, and nanochannel-open VMSF/ITO electrodes were employed as working electrodes. Figure 2c,d show the CV curves of Fe(CN)_6_^3−^ and Ru(NH_3_)_6_^3+^ on the two types of electrodes, respectively. The results indicated that no significant CV signals were detected on the SM@VMSF/ITO electrodes in either probe solution. This was attributed to the presence of micelles, which hindered the diffusion of Fe(CN)_6_^3−^ and Ru(NH_3_)_6_^3+^ molecules from the solution to the electrode surface, confirming the integrity and crack-free nature of the prepared VMSF film. Furthermore, compared with the bare ITO electrodes, the VMSF/ITO electrodes showed a decreased signal in the Fe(CN)_6_^3−^ solution and an increased signal in the Ru(NH_3_)_6_^3+^ solution. This phenomenon was due to the negatively charged silanol groups (p*K*a~2) present on the VMSF surface under the experimental conditions, which exhibited an attractive effect on the positively charged probes and a repulsive effect on the negatively charged probes. Thus, the VMSF nanochannels repelled the negatively charged probes while enriching the positively charged ones. This observation clearly demonstrated the charge selectivity of the VMSF/ITO electrode. These results demonstrated that the prepared VMSF film was intact and crack-free, exhibiting significant enrichment ability for positively charged probe molecules. These findings provide valuable insights for the construction of homogeneous aptasensors using cationic ECL emitter.

### 2.3. Characterization of Graphene Oxide

**Figure 3:**
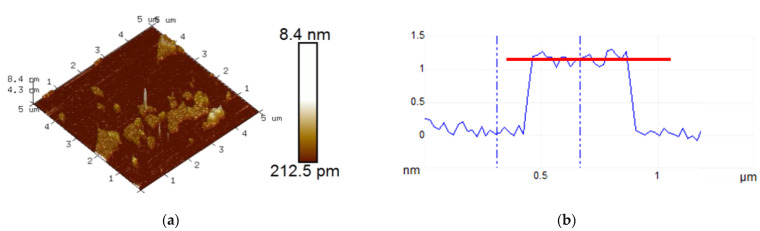

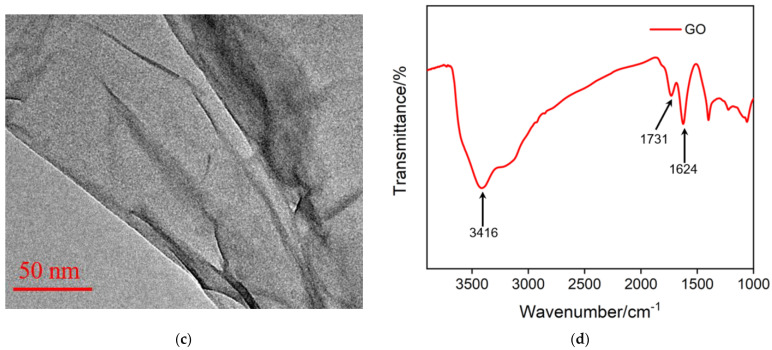
AFM image (**a**), the height-distance line (**b**), TEM image (**c**) and FT-IR spectrum (**d**) of GO.

Graphene oxide (GO) was used in this study as the material for immobilizing the recognition aptamer and for adsorbing the ECL emitter, as well as the redox probe Ru(bpy)_3_^2+^ to construct a 2D composite probe. Figure 3a show the atomic force microscopy (AFM) image of GO. It can be observed that the thickness of the GO nanosheet was approximately 1.2 nm, indicating a monolayer graphene structure (Figure 3b). The transmission electron microscopy (TEM) image in Figure 3c further confirmed the layered structure of GO. Figure 3d presents the Fourier transform infrared (FT-IR) spectrum of GO, where characteristic peaks of O-H stretching vibration (3416 cm^−1^), C=O stretching vibration (1731 cm^−1^), sp^2^ carbon vibration (1625 cm^−1^), C-O-C stretching vibration (1300 cm^−1^), and C-O stretching vibration (1050 cm^−1^) were observed, confirming the presence of oxygen-containing groups on the surface of GO.

### 2.4. Feasibility of 2D Composite Probe for Homogeneous Sensor and EC/ECL Dual-Mode Detection

**Figure 4:**
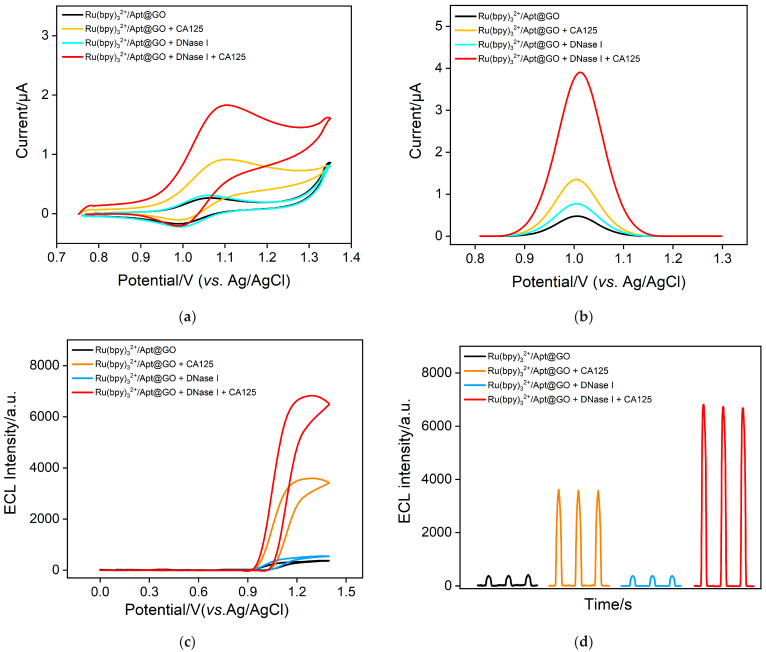
(**a**) CV, (**b**) DPV curves, and (**c**) ECL intensity-potential curves obtained on VMSF/ITO. ECL-potential, (**d**) ECL-time curve obtained on VMSF/ITO with Ru(bpy)_3_^2+^/Apt@GO composite probe and different substances.

Ru(bpy)_3_^2+^ not only functions as an ECL probe but also serves as a redox probe. The feasibility of constructing a homogeneous aptasensor based on the 2D composite probe for the EC/ECL dual-mode detection of CA125 was investigated. The results obtained from CV and DPV tests are shown in Figure 4a,b. In the Ru(bpy)_3_^2+^/Apt@GO 2D probe solution, only a very low background signal was observed (black line). This is primarily due to the VSMF having extremely small nanochannels, which exhibited a significant size exclusion effect, thereby hindering the diffusion of the 2D composite probe and preventing the detection of Ru(bpy)_3_^2+^ electrochemical signals. After the addition of the nuclease DNase I, the CA125 aptamer bound to the GO surface, where GO effectively protected the aptamer from cleavage. As a result, the electrochemical signal remained low and showed minimal change compared with the signal before DNase I addition (blue line). However, when CA125 was introduced into the 2D composite probe, the electrochemical signal response increased significantly (red line). This was attributed to the specific interaction between the aptamer and CA125, forming the aptamer-CA125 complex. This complex detached from the GO surface into the solution, leading to the release of a large amount of Ru(bpy)_3_^2+^ molecules, which were originally adsorbed by GO and the aptamer. Consequently, the concentration of free probe molecules in the solution increased, and positively charged Ru(bpy)_3_^2+^ molecules were enriched on the negatively charged nanochannels, generating a significantly enhanced signal. In addition, the ECL signal was also verified (Figure 4c,d). In the presence of the co-reactant TPA, the ECL signal of Ru(bpy)_3_^2+^ can be triggered under specific electrochemical conditions. Specifically, during the positive potential scan, TPA and Ru(bpy)_3_^2+^ were oxidized at the electrode to form TPA radicals and Ru(bpy)_3_^3+^. The TPA radicals then underwent a redox reaction with Ru(bpy)_3_^3+^, generating an excited state of Ru(bpy)_3_^2+^*, which emitted light when it returned to the ground state.

### 2.5. Optimization of Conditions

**Figure 5:**
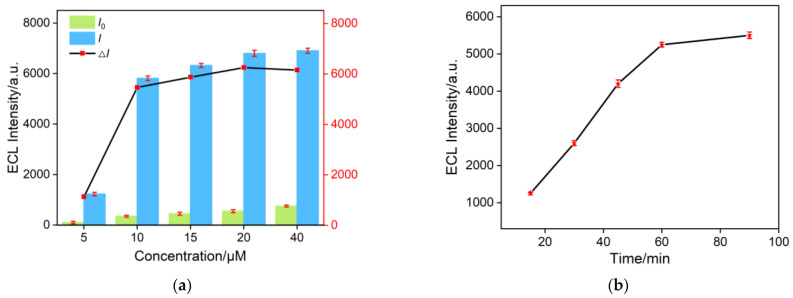
(**a**) Optimization of concentration of Ru(bpy)_3_Cl_2_. (**b**) ECL intensity obtained using different reaction times with DNase I. The star symbol represented the date point.

By adjusting the concentration of Ru(bpy)_3_^2+^, its adsorption on GO and the aptamer can be gradually saturated, resulting in the maximum ECL intensity change (△*I* = *I* − *I*_0_, where *I*_0_ and *I* represented the ECL intensity before and after the addition of CA125, respectively) upon addition of the target CA 125. As shown in Figure 5a, in the solution without CA125, the system maintained a low ECL signal (*I*_0_). The ECL response increased after the specific recognition of the aptamer to the target CA125, followed by the cyclic cleavage action of DNase I. Considering both a low background signal and a high ECL signal change, 20 μM Ru(bpy)_3_^2+^ was selected as the optimal concentration for fabricating the 2D composite probe. As DNase I acted as a signal amplifier, its cleavage time significantly impacted the detection sensitivity. Thus, the incubation time of DNase I with the aptamer was optimized. The ECL signal intensity after different cleavage times was recorded, as shown in Figure 5b. As shown, long DNase I reaction time facilitated the dissociation of the aptamer complex. After 60 min of reaction, the ECL signal was not significantly changed. Thus, a 60 min enzyme reaction time was chosen as the optimal detection condition.

### 2.6. ECL Detection of CA 125

**Figure 6:**
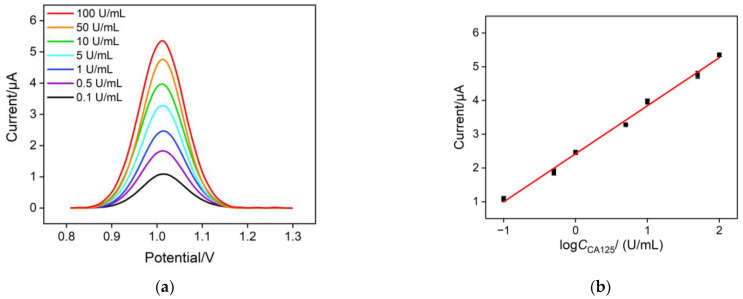

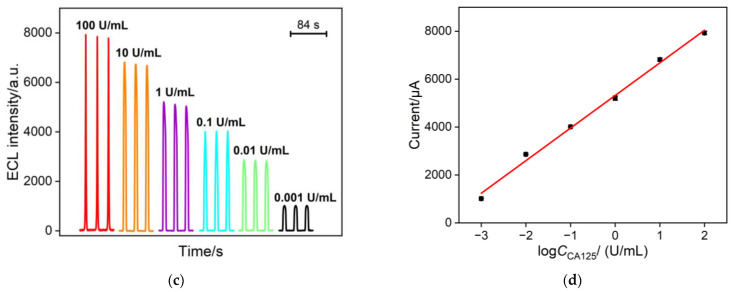
(**a**) DPV curves obtained in the presence of different concentrations of CA125 (0.1 U/mL, 0.5 U/mL, 1 U/mL, 5 U/mL, 10 U/mL, 50 U/mL, and 100 U/mL), and (**b**) the corresponding linear regression curves. (**c**) ECL intensity−time response obtained with different concentrations of CA125 (0.001 U/mL, 0.01 U/mL, 0.1 U/mL, 1 U/mL, 10 U/mL, and 100 U/mL), and (**d**) the corresponding linear regression curve.

Under the optimal conditions, the 2D composite probe Ru(bpy)_3_^2+^/Apt@GO was incubated with different concentrations of CA125 and an equivalent amount of DNase I for 60 min. The signal of Ru(bpy)_3_^2+^ was then detected using both electrochemical and electrochemiluminescence (ECL) methods (Figure 6). In the electrochemical detection mode, the DPV response curve of the sensing electrode for CA125 detection is shown in Figure 6a. As the CA125 concentration increased, the anodic peak current increased significantly. Figure 6b displays the linear regression curve between the DPV peak current and the logarithm of the CA125 concentration (log*C*_CA125_). When the CA125 concentration ranged from 0.1 to 100 U/mL, the DPV peak current exhibited a linear relationship with log*C*_CA125_ (*I*_EC_ = 1.42 ± 0.0511 log*C*_CA125_ + 2.42 ± 0.0439, *R*^2^ = 0.994, Figure 6b). The detection limit (LOD), calculated using a signal-to-noise ratio (S/N) of 3, was found to be 91 mU/mL. In the ECL detection mode, the ECL intensity (*I*_ECL_) measured at different CA125 concentrations is shown in Figure 6c. When the CA125 concentration ranged from 0.001 to 100 U/mL, the ECL intensity exhibited a linear relationship with log*C*_CA125_ (*I*_ECL_ = 1360 ± 48.7 log*C*_CA125_ + 5319 ± 86.62, *R*^2^ = 0.994, Figure 6d). The LOD was 0.4 mU/mL (S/N = 3).

### 2.7. Selectivity of the Aptasensor

**Figure 7:**
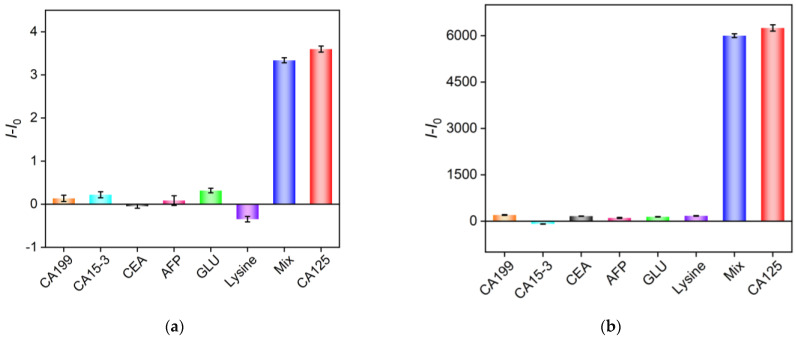
Difference in DPV peak current (**a**) and ECL signal (**b**) response (*I* − *I*_0_) without (*I*_0_) or with (*I*) CA125. During incubation, CA199 (10 U/mL), CA 15-3 (10 U/mL), CEA (500 ng/mL), AFP (500 ng/mL), Glu (50 μM), lysine (50 μM), or CA125 (10 U/mL) was added or mixed.

To investigate the selectivity of the homogeneous aptasensor for detecting CA125, several other potential interfering substances, including tumor biomarkers such as carbohydrate antigen 199 (CA199), carbohydrate antigen 153 (CA15-3), carcinoembryonic antigen (CEA), alpha-fetoprotein (AFP), glucose (Glu), and lysine, were examined. Figure 7a,b show the signal responses in both EC and ECL modes when each of these substances, CA125, and a mixture of CA125 with the other substances were presented. It can be observed that only CA125 induced the release of the Ru(bpy)_3_^2+^ probe, leading to a significant increase in both EC and ECL signals, while the other substances caused negligible signal changes. These results demonstrate that the constructed aptasensor exhibited excellent selectivity for detecting CA125.

### 2.8. Analysis of Real Samples

To verify the practical applicability of the fabricated aptasensor, the standard addition method was used to measure CA125 in fetal bovine serum samples. A series of known concentrations of CA125 were added to the serum samples, which were then diluted 50-fold with PBS, followed by EC or ECL detection. The results, shown in Table 1, indicated that both EC and ECL detection modes provided satisfactory recovery rates, ranging from 94.5% to 109%. The relative standard deviation (RSD) of the three measurements was no more than 3.6%, demonstrating high detection accuracy. Due to the simplicity and ease of operation of the detection process, the developed homogeneous aptasensor shows great potential for real sample analysis [33,34].

## 3. Materials and Methods

### 3.1. Chemicals and Materials

Tetraethyl orthosilicate (TEOS), disodium hydrogen phosphate, glucose, potassium ferrocyanide, sodium dihydrogen phosphate, potassium ferrocyanide, and cetyl trimethylammonium bromide (CTAB) were purchased from Aladdin Bio-Chem Technology Co., Ltd. (Shanghai, China). Tris(2,2′-bipyridyl)ruthenium(II) chloride (Ru(bpy)_3_Cl_2_) was obtained from Sigma-Aldrich (Shanghai, China). All chemicals and reagents were of analytical grade and were used without further purification. Nuclease I was obtained from Thermo Scientific, and carcinoembryonic antigen (CEA), carbohydrate antigen 15-3 (CA15-3), carbohydrate antigen 125 (CA125), and carbohydrate antigen 19-9 (CA19-9) were purchased from Kaiji Biotechnology Co., Ltd. (Beijing, China). The carbohydrate antigen 125 (CA 125) aptamer, 5′-CTCACTATAGGGAGACAAGAATAAACGCTCAA-3′, was synthesized by Shenggong Biotechnology Co., Ltd. (Shanghai, China). Indium tin oxide (ITO) conductive glass electrodes (sheet resistance <17 Ω/square resistance, thickness 100 ± 20 nm) were obtained from Kawei Optoelectronics Technology Co., Ltd. (Zhuhai, China). Prior to use, ITO electrodes (0.5 cm × 0.5 cm) were soaked overnight in 1 M NaOH. The electrodes were then ultrasonically cleaned with acetone, ethanol, and ultrapure water, respectively.

### 3.2. Measurements and Instrumentations

The surface morphology of GO was characterized using a Bruker Dimension Icon atomic force microscope (AFM, Saarbrücken, Germany). Electrochemiluminescence (ECL) measurements were performed using the MPI-E II analysis system from Xi’an Ruimait Analytical Instrument Co., Ltd. (Xi’an, China). Electrochemical (EC) measurements, including cyclic voltammetry (CV) and differential pulse voltammetry (DPV), were conducted on a PGSTAT302N electrochemical workstation from Autolab (Metrohm, Heilissau, Switzerland). ECL and EC measurements were carried out using a traditional three-electrode system, with the working electrode, platinum wire, or platinum plate as the counter electrode and an Ag/AgCl electrode (saturated KCl solution) as the reference electrode. The morphology of VMSF and modified electrodes was studied using a transmission electron microscope (TEM, HT7700, Hitachi, Tokyo, Japan) and scanning electron microscope (SEM, SU8010, Hitachi, Tokyo, Japan). The acceleration voltage for TEM measurements was 100 kV. The VMSF was scraped from the VMSF/ITO surface using a blade, then dispersed in ethanol via ultrasonication and dropped onto a copper grid for testing. The acceleration voltage for SEM measurements was 5 kV. Prior to testing, the samples were gold-coated.

### 3.3. Preparation of VMSF-Modified ITO Electrode

VMSF were grown on ITO substrates using the electrochemical-assisted self-assembly (EASA) method [47,48,49]. The precursor solution for growing VMSF consisted of ethanol (20 mL), NaNO_3_ (20 mL, 0.1 M, pH = 2.6), TEOS (2.833 g), and CTAB (1.585 g), which was stirred for 2.5 h before use. The cleaned ITO electrode was then immersed in the precursor solution, and a current density of −0.7 mA/cm^2^ was applied for 10 s to obtain an electrode containing surfactant micelles (SMs) in the nanochannels, labeled as SM@VMSF/ITO. The resulting SM@VMSF/ITO electrode was then stirred in a 0.1 M HCl-ethanol solution for 5 min to remove SM, yielding the VMSF/ITO electrode with open nanochannels.

### 3.4. Preparation of the Composite Probe Ru(bpy)_3_^2+^/Apt@GO

GO (2 mg/mL) and CA125 aptamer (Apt, 100 μM) were added to a Ru(bpy)_3_^2+^ solution (20 μM) and ultrasonically mixed for 1 h. The mixture was then centrifuged at 5000 rpm for 30 min. The resulting precipitate was washed three times with phosphate-buffered saline (PBS, 0.01 M, pH = 7.4) and redispersed in PBS for further use.

### 3.5. EC/ECL Dual-Mode Detection of CA125

Different concentrations of CA125 and 20 U of DNase I were added to the Ru(bpy)_3_^2+^/Apt@GO probe solution and incubated for 1 h at 37 °C. For ECL measurement, VMSF/ITO was used as the working electrode. After incubation, 3 mM tripropylamine (TPA) was added to the solution, and the ECL test was triggered by a continuous CV process, with ECL signals recorded simultaneously. The potential range was set from 0 to 1.4 V, with a scan rate of 100 mV/s. The photomultiplier tube (PMT) voltage was set to 400 V. For EC measurement, the VMSF/ITO electrode was immersed in the incubation solution for 5 min, followed by recording the DPV curve. For real sample analysis, the standard addition method was used to measure CA125 in fetal bovine serum (FBS). Prior to measurement, the sample was diluted 20 times with PBS.

## 4. Conclusions

In this work, a homogeneous aptasensor was constructed using a 2D composite probe, utilizing the VMSF nanochannel array to achieve size exclusion of the 2D composite probe, electrostatic enrichment of the cationic probe Ru(bpy)_3_^2+^ with both redox and ECL properties, and signal amplification through cyclic cleavage by DNase I. This enabled the dual-mode EC/ECL detection of CA125. The 2D composite probe combined the recognition aptamer and Ru(bpy)_3_^2+^ on graphene oxide (GO), and the aptamer specifically recognized the target CA125 to form the aptamer-CA125 complex. DNase I cleaved the aptamer-CA125 complex, releasing CA125 and simultaneously causing Ru(bpy)_3_^2+^ to detach and enter the solution. The electrostatic enrichment of the cationic Ru(bpy)_3_^2+^ on the VMSF nanochannels enhanced both the EC and ECL signals. The sensor exhibited high detection selectivity. Owing to the convenient preparation of the 2D composite probe and simple detection electrode, the developed aptasensor shows great potential for tumor biomarker detection.

## Figures and Tables

**Table 1:** EC and ECL detection of CA125 by standard addition method using the developed homogenous aptasensor.

Sample	Detection Mode	Added (ng mL^−1^)	Found (ng mL^−1^)	RSD (%, n = 3)	Recovery (%)
Fetal bovine serum ^a^	EC	0.500	0.48	3.6	96.0
	EC	5.00	5.46	2.9	109
	EC	50.0	52.3	2.1	105
	ECL	0.100	0.0945	1.2	94.5
	ECL	1.00	1.09	1.5	109
	ECL	10.0	9.45	1.1	94.5

^a^ The fetal bovine serum with added CA 125 was diluted by 50 times using PBS (0.01 M, pH 7.4) before detection.

## Data Availability

The data presented in this study are available on request from the corresponding author.
